# Morphological association between the muscles and bones in the craniofacial region

**DOI:** 10.1371/journal.pone.0227301

**Published:** 2020-01-10

**Authors:** Masahito Yamamoto, Hiromasa Takada, Satoshi Ishizuka, Kei Kitamura, Juhee Jeong, Masaki Sato, Nobuyuki Hinata, Shinichi Abe

**Affiliations:** 1 Department of Anatomy, Tokyo Dental College, Tokyo, Japan; 2 Tokyo Dental College Research Branding Project, Tokyo Dental College, Tokyo, Japan; 3 Department of Histology and Developmental Biology, Tokyo Dental College, Tokyo, Japan; 4 Department of Basic Science and Craniofacial Biology, New York University College of Dentistry, New York, NY, United States of America; 5 Laboratory of Biology, Tokyo Dental College, Tokyo, Japan; 6 Department of Urology, Kobe University Graduate School of Medicine, Hyogo, Japan; University of Minnesota Medical School, UNITED STATES

## Abstract

The strains of inbred laboratory mice are isogenic and homogeneous for over 98.6% of their genomes. However, geometric morphometric studies have demonstrated clear differences among the skull shapes of various mice strains. The question now arises: why are skull shapes different among the mice strains? Epigenetic processes, such as morphological interaction between the muscles and bones, may cause differences in the skull shapes among various mice strains. To test these predictions, the objective of this study is to examine the morphological association between a specific part of the skull and its adjacent muscle. We examined C57BL6J, BALB/cA, and ICR mice on embryonic days (E) 12.5 and 16.5 as well as on postnatal days (P) 0, 10, and 90. As a result, we found morphological differences between C57BL6J and BALB/cA mice with respect to the inferior spine of the hypophyseal cartilage or basisphenoid (SP) and the tensor veli palatini muscle (TVP) during the prenatal and postnatal periods. There was a morphological correlation between the SP and the TVP in the C57BL6J, BALB/cA, and ICR mice during E15 and P0. However, there were not correlation between the TVP and the SP during P10. After discectomy, bone deformation was associated with a change in the shape of the adjacent muscle. Therefore, epigenetic modifications linked to the interaction between the muscles and bones might occur easily during the prenatal period, and inflammation seems to allow epigenetic modifications between the two to occur.

## Introduction

To date, over 450 inbred mouse strains have been described and developed, hence providing abundant phenotypes and genomic backgrounds for genetic studies. Most inbred laboratory strains have originated from a limited founder population of *Mus musculus* and *M*. *m*. *domesticus* housed within a few research facilities and laboratories [1]. Most of these strains have been bred for over 150 generations and are isogenic and homogeneous for over 98.6% of their genomes [2]. Nevertheless, geometric morphometric studies have revealed clear differences with respect to the skull shapes of various mice strains [3, 4]. However, the underlying reasons for the differences in the skull shapes among various mice strains remain unclear.

Ever since Charles Darwin published his theory of evolution in the book titled *On the Origin of Species* in 1859, researchers in the field of evolutionary developmental biology have sought to unravel the mechanisms of evolution of phenotypic variations. By the end of the 20th century, the researchers had identified the specific genes and allelic variants that adapt to phenotypic variations [5]. However, genotypic characterization alone does not explain all the phenotypic variations in natural populations. In 1940–1950, before the golden age of evolutionary genetics, Conrad Waddington had already described the extragenetic factors that contribute to phenotypic variations [6, 7]. He demonstrated that embryo fruit flies reared in a high-temperature environment have different wing structures than their counterparts in the control group. Subsequently, he selectively bred the fruit flies that displayed the new characteristics for several generations. Consequently, the progeny displayed the new characteristics even in the absence of the environmental stimulus. He defined this phenomenon as “Epigenetics,” which refers to the effect of internal and external interactions between the environment and genes on the evolution of the phenotype [6, 7].

The term epigenetics encompasses the tissue–tissue interactions, such as the effect of the muscle on the bone during their development and maintenance periods. The biomechanical interaction between the muscle and bone has typically been investigated in three types of studies, i.e., analyses of the correlation between muscle volume and bone size [8–11], comparisons of skull shapes between mice feeding on hard and soft diets [12, 13], and study of the effects of muscle atrophy on bone shapes [13, 14]. As with the muscle–bone interaction in adults, the bone shape is associated with the muscle-induced loading during the embryonic development period. Sharir et al. [15] showed that mice paralyzed due to muscular dysgenesis exhibit abnormal circular-shaped long bone diaphysis, which indicated the effect of *in utero* muscle load on bone development. Similar aberrant development of the shape of long bones was also observed in the studies of mice without muscles [16–18]. Particularly, Rot-Nikcevic et al. [19] described that muscle defects had a noticeable effect on the morphology of the mandible. However, few studies have focused on the morphological interaction between the muscle and bone.

Illustrations and photographs in the anatomical textbooks show that skeletal muscles properly fit onto the surfaces of adjacent bone and other skeletal muscles. This is clearly observed in the cross-sectional images of the arm, thigh, head, or neck [20]. Moreover, our previous studies have suggested that muscle anlagen are already fitted onto the surface of the adjacent bone or cartilage analgen during the prenatal development [21–27]. Therefore, the interaction between the muscle and bone may affect their shape during the developmental period because the skeletal muscles properly fit onto the surfaces of adjacent bone for a lifetime, including in the fetal period [21–27].

Our findings showed differences between C57BL6J and BALB/cA mice with respect to the shape of a specific part of the skull and its adjacent muscle during the prenatal and postnatal periods. Differences in the shape of the skull and its surrounding muscles among the mice strains may result from epigenetic processes linked to the interaction between their adjacent parts. To test these predictions, we examined the morphological associations between the skull and its adjacent muscles.

## Materials and methods

### Ethics statement

All experiments related to mice were performed in accordance with the National Institutes of Health guidelines for care and use of animals. In addition, these experiments were also approved by the Tokyo Dental College Institutional Animal Care and Use Committee (IACUC) (protocol #240106).

### Study design

We employed C57BL6J and BALB/cA mice on the embryonic days (E) 12.5 and 15.5 and on the postnatal days (P) 0, and 10, respectively. In total, 40 C57BL6J and BALB/cA mice were used in this study. To examine the morphological association between a specific part of the skull and its adjacent muscle in detail, we also used not only 40 C57BL6J and BALB/cA mice but 10 ICR mice (E15.5, P0 and P10). In all timed pregnancies, the date of development of the vaginal plug was defined as E0.5. For the harvesting of embryos, timed-pregnant females were sacrificed by CO_2_ intoxication. The gravid uterus was dissected out and suspended in a bath of cold phosphate-buffered saline, and the embryos were harvested after amnionectomy and removal of placenta. The postnatal mice were also euthanatized by using CO_2_ intoxication. All experiments involving mice were approved by the IACUC Committee at the Tokyo Dental College.

### Histological analysis and three-dimensional reconstruction

**Fig 1:**
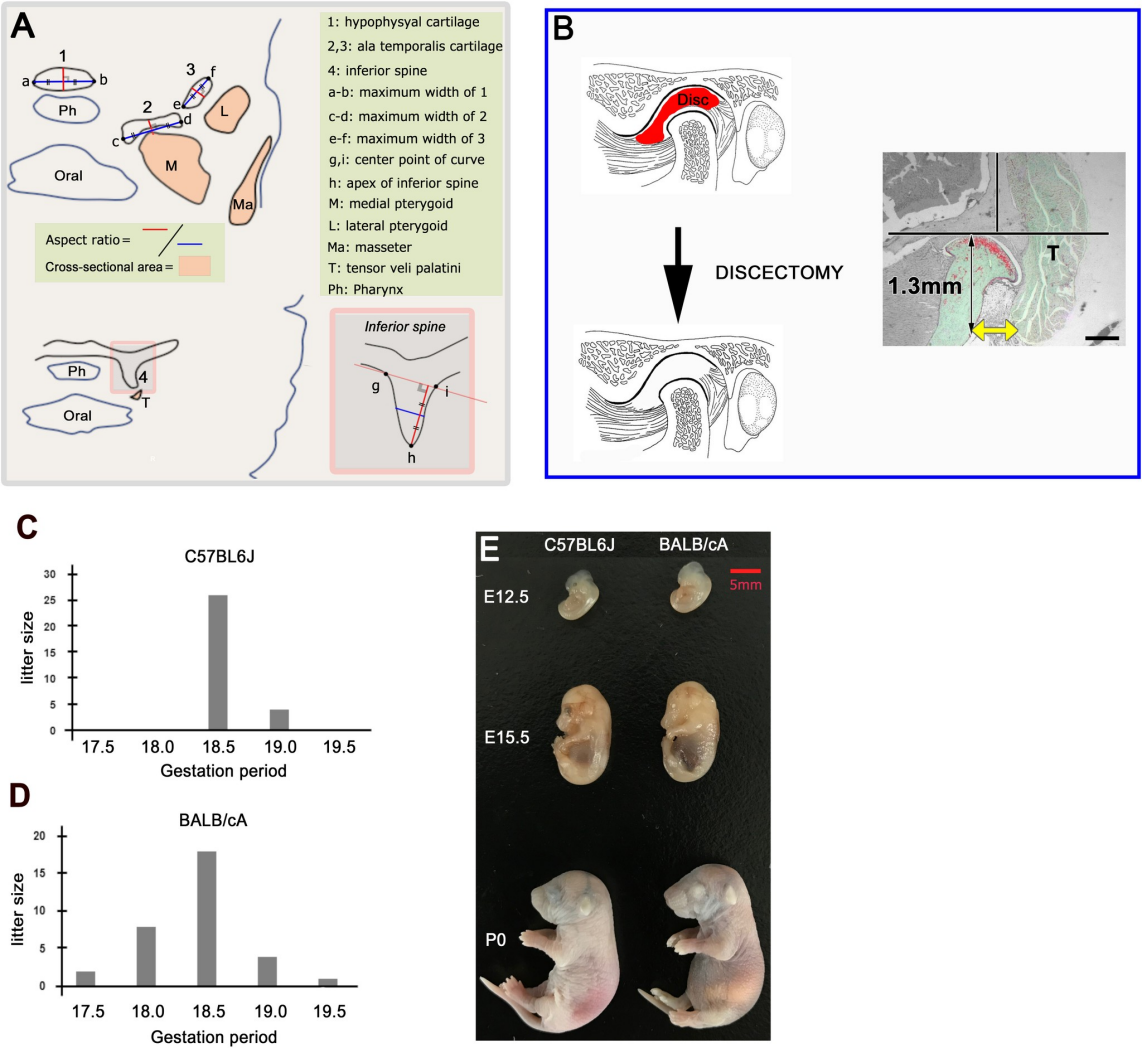
Study design. Panel A: Schema of frontal sections for morphometric analysis of the middle cranial base. The parameters are shown in the schema. Panel B: A schematic illustration of discectomy. The measurement point of the condylar neck as 1.3 mm inferior to the top of the condylar head. The distance between the condylar neck and the temporalis muscle (yellow arrow). Panels C and D: The mean gestation in C57BL6J and BALB/cA mice was 18.5 days. Panel E: Crown-rump length (CRL) at E12.5, E15.5, and P0. There is no significant difference between the two strains in this respect (Table 1).

Before being subjected to demineralization with 10% ethylenediaminetetraacetic acid, all the mice were fixed in 4% phosphate-buffered paraformaldehyde. Next, the paraffin blocks were prepared by using standard methods, and a series of 5- to 10-μm-thick tissue sections were cut by a sliding microtome. Then, we prepared the frontal sections, followed by staining with hematoxylin and eosin (H&E). For the morphometric analysis of the middle cranial base and its surrounding muscles, the frontal sections, which included this area, were prepared, and the parameters were calculated (Fig 1A). These parameters were measured by using the ImageJ software (National Institutes of Health).

**Table 1:** Crown-rump length of mice (mean ± SD).

	C57BL6J	BALB/cA	*P-value*
E12.5 (mm)	6.12±0.34	6.21±0.31	*P = 0*.*18*
E15.5 (mm)	12.69±0.64	12.25±0.66	*P = 0*.*45*
P0 (mm)	22.97±0.95	23.18±1.06	*P = 0*.*62*

For three-dimensional (3D) reconstruction, we loaded the digital images of the H&E stained serial sections into Amira (Visage Imaging, Inc.) by using a voxel size that was appropriate for the section thickness.

### Immunohistochemical analysis

Sections were incubated overnight at 4°C with the following primary antibodies: mouse anti-desmin antibody (1:1000, Merck Millipore) and rabbit anti-sox9 antibody (1:1000, Merck Millipore). Afterwards, they were stained at the room temperature for 1.5 h with the following secondary antibodies: donkey anti-mouse IgG Alexa Fluor 488 (1:1000, Thermo Fisher Scientific) and donkey anti-goat IgG Alexa Fluor 555 (1:1000, Thermo Fisher Scientific).

### Partial discectomy of the temporomandibular joint

Four C57BL6J mice (age, 3 months) were used. Each mouse was anesthetized with intra-peritoneal 70 μg ketamine and 15 μg xylazine per microgram of bodyweight. Incisions were made over the right temporomandibular joint and through the subcutaneous and masseter muscle layers to allow the removal of a part of the disk (Fig 1B) [28, 29]. The left temporomandibular joint, which did not undergo surgery, was used as a control. At 70 days after discectomy, the mice were sacrificed by CO_2_ intoxication. Before demineralization with 10% ethylenediaminetetraacetic acid, all the mice were fixed in 4% phosphate-buffered paraformaldehyde. Paraffin blocks were prepared by using standard methods, and a series of 5- to 10-μm-thick tissue sections were cut by a sliding microtome. Finally, we prepared the frontal sections that were stained with safranin O/fast green [28, 29].

We measured the distance between the condylar neck and the temporalis muscle. We determined a 1.3-mm inferior point from the top of the condylar head as the measurement point of the condylar neck (Fig 1B). The vertical line to the squamous part of the temporal bone was determined as the reference line (Fig 1B). These parameters were measured by using the ImageJ software (National Institutes of Health).

### The observation of osseous morphology by 3-D reconstruction

Four C57BL6J mice (age, 3 months) were used. The materials were imaged by using a micro-CT system (HMX‐225Actis4; Tesco Co, Tokyo, Japan) and following the basic conditions: tube voltage, 100 kV; tube current, 120 μA; slice width, 50 μm; matrix size, 512 × 512; and slice voxel size, 52.7 × 52.7 × 50 μm. The 3D reconstructions were obtained from the slice images by using the 3D reconstruction software (VG Studio, Volume Graphics, Heidelberg, Germany) for the observation of osseous morphology.

### Statistical analysis

*P*-values were calculated by using the Student’s *t*-test. Between-group differences associated with the *p*-values of <0.05 were considered statistically significant (*p < 0.05, **p < 0.01, and ***p < 0.001 are used throughout the paper). Error bars show the standard deviation of the mean. All statistical analyses were performed by using SPSS 21.0 (IBM, Armonk, NY, USA).

The Spearman correlation coefficient (r) was calculated to determine the linear association between the cross-sectional area (CSA) of the tensor veli palatini muscle (TVP) or the TVP angle and aspect ratio of the inferior spine of the hypophyseal cartilage or basisphenoid (SP). The outcomes were interpreted according to the degree of association as strong (0.7–1), moderate (0.5–0.7), or low (0.3–0.5) after considering the significant correlation (p < 0.05) values.

## Results

### Comparative anatomy of two mice strains (C57BL6J vs Balb/cA)

The gestations of the C57BL6J and BALB/cA mice averaged 18.5 days (Fig 1C and 1D). We compared the crown-rump lengths of both C57BL6J mice and BALB/cA. There was no significant difference between the two strains (Table 1 and Fig 1E).

**Fig 2:**
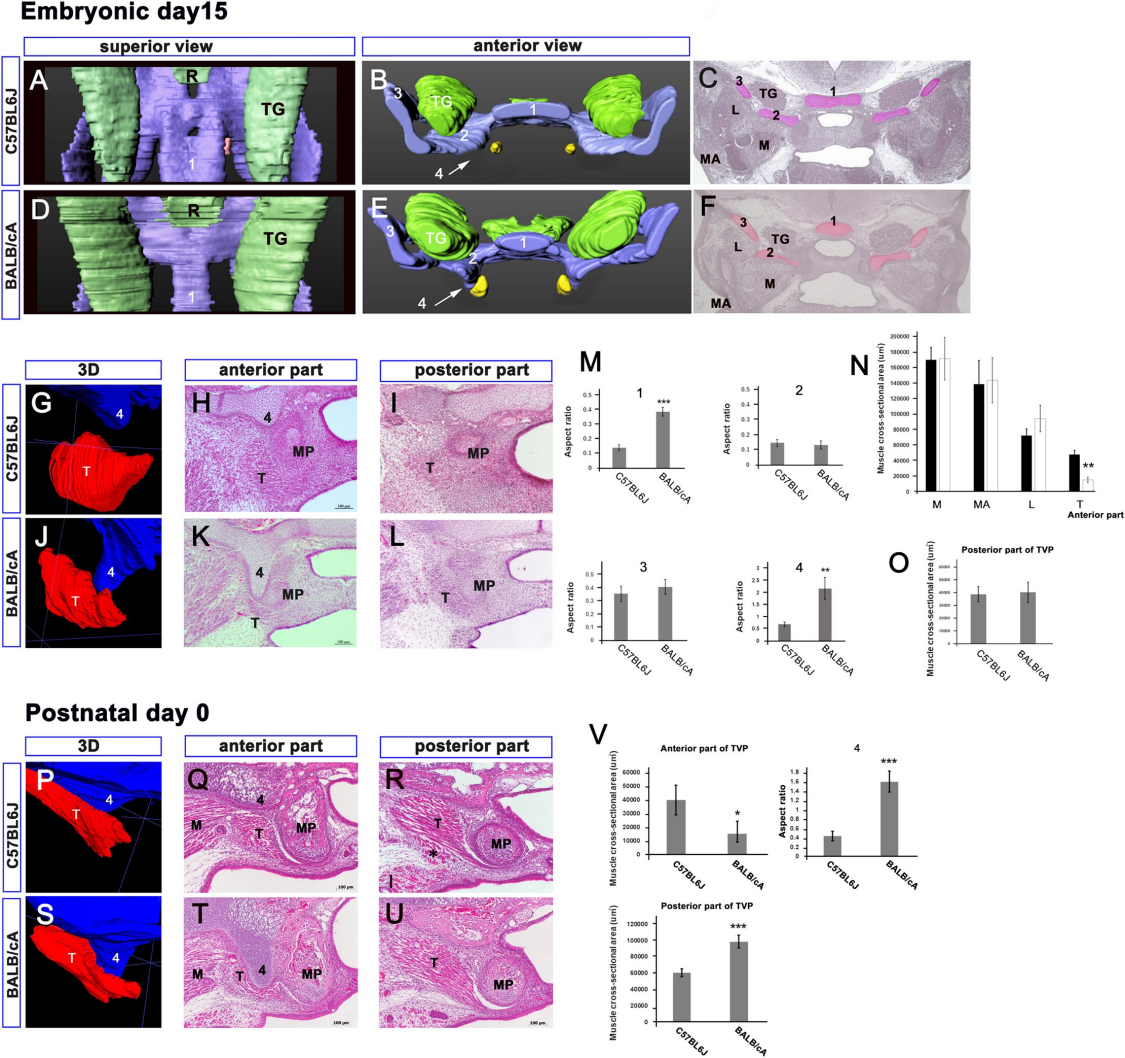
Comparative anatomy of two mice strains at E15 and P0. Panels A and D show the superior view of the middle cranial base, and the lower side of the figures correspond to the anterior side of the head. Panels B and E show the anterior view of the middle cranial base. Panels C and F are frontal sections. Panels G-L and P-U show high magnification views, including the inferior part of the hypophyseal cartilage (4) and tensor veli palatini (T). Morphological differences are apparent in the middle cranial base of the two mouse strains at E15.5 and P0 (panels A-L, P-U). The inferior part of the hypophyseal cartilage (4) has a small spine that is longer in BALB/cA mice than that in C57BL6J (panels G, J, M, P, S, and V). The anterior part of the tensor veli palatini (T) in BALB/cA mice is smaller than that in C57BL6J (panels H, K, N, Q, T, and V).1. Anterior part of the hypophyseal cartilage; 2, 3. ala temporalis cartilage; 4. Inferior part of the hypophyseal cartilage. M, masseter; MA, medial pterygoid; L, lateral ptergoid; T, tensor veli palatine; TG, trigeminal ganglion; asterisk. newly formed muscle. Scale bar = 500 μm (panels C, F), 100 μm (panels H, I, K, L, Q, R, T, U).

The middle cranial base comprised the trabecular, hypophyseal, and ala temporalis cartilages at E15.5 [30]. The hypophyseal cartilage (1) was narrower in the BALB/cA mice than that in the C57BL6J mice (*P <* 0.001) (Fig 2A–2F and 2M). The ala temporalis did not differ between the C57BL6J and BALB/cA mice (medial part (2), *P =* 0.36; lateral part (3), *P =* 0.42) (Fig 2A–2F and 2M). The SP (4) was longer in the BALB/cA mice than in the C57BL6J mice (*P < 0*.*01*) (Fig 2G, 2J and 2M). The CSAs of the masseter, medial pterygoid, and lateral pterygoid were found to be comparable between the two mice strains (Fig 2C, 2F and 2N). The CSA of the anterior part of the TVP was smaller in the BALB/cA mice than that in the C57BL6J mice (*P <* 0.01) (Fig 2H, 2K and 2N). The CSA of the posterior part did not differ between the strains (*P* = 0.77) (Fig 2I, 2L and 2O).

On P0, longer SPs (4) were identified in the BALB/cA mice, whereas the C57BL6J mice had shorter SPs (4) (*P* < 0.001) (Fig 2P, 2S and 2V). The anterior TVP in the C57BL6J mice had a larger CSA than that in the BALB/cA mice (*P* < 0.05) (Fig 2Q, 2T and 2V). A newly formed muscle bundle was only identified inferior to the posterior part of the TVP in the C57BL6J mice (Fig 2R), and the two muscles were in contact with each other. Differences in CSAs were apparent in the posterior TVPs of each strain (*P* < 0.001) (Fig 2R, 2U and 2V).

**Fig 3:**
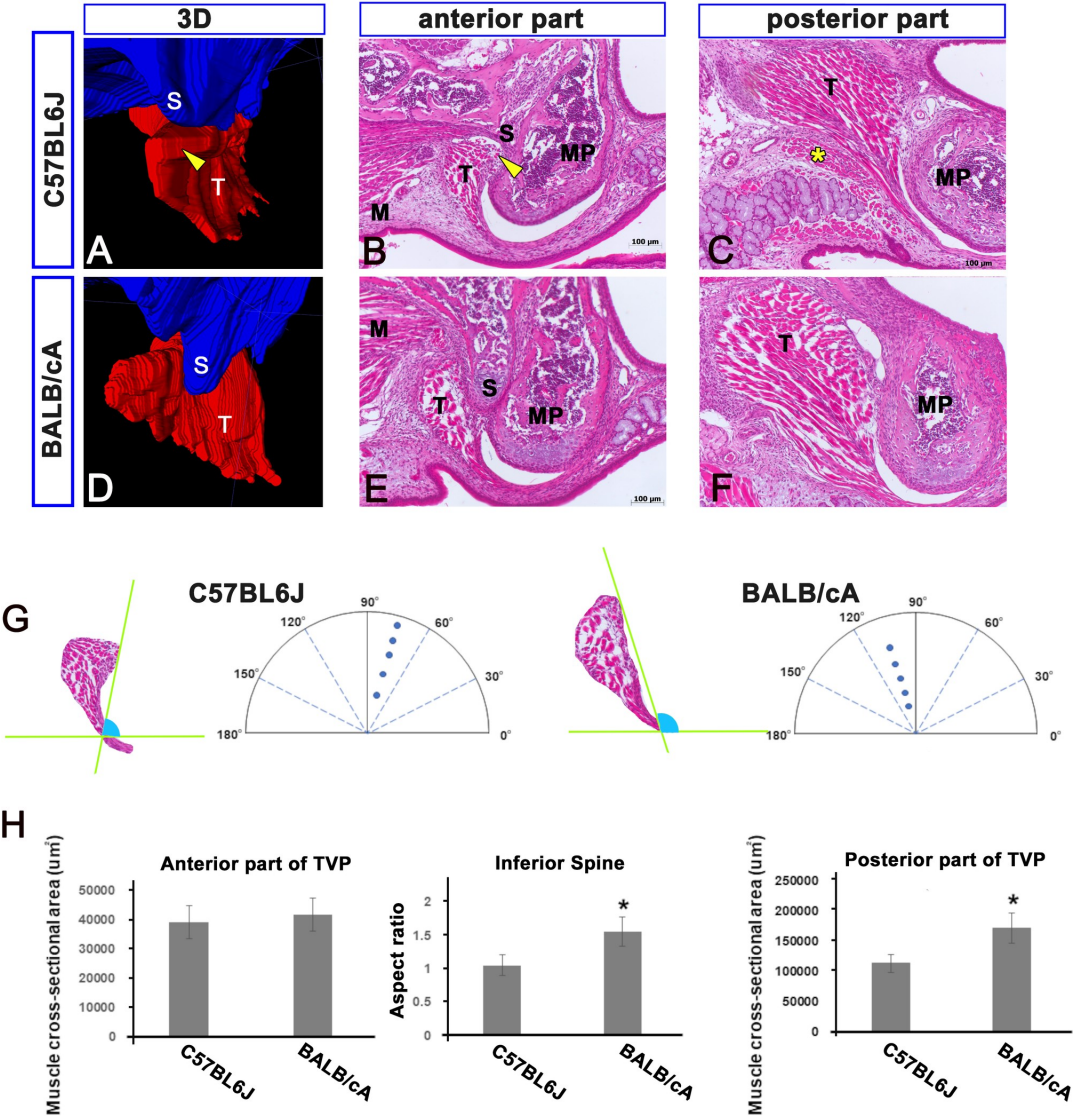
Comparative anatomy of two mice strains at P10. All panels show high magnification views including the inferior part of the hypophyseal cartilage (S) and the tensor veli palatini (T). Panels B and E (C and F) show the anterior (posterior) part of the TVP. The TVP angle between the line connecting the most inferior point with the superomedial point and a horizontal line are different between the C57BL6J mice (74.3° ± 3.0°) and BALB/cA mice (105.2° ± 5.2°) (panels A, B, D, E, and G; arrow heads). The shapes of the inferior spine (S) show differences (panels B, E, and H). Although the cross-sectional areas (CSAs) of the anterior TVPs show no differences (panels B, E, and H), the CSAs of the posterior TVPs differed significantly (panel C, F, and H).M. medial pterygoid; MP. medial pterygoid process; S. inferior spine of the hypophyseal cartilage; T. tensor veli palatini, asterisk. newly formed muscle. Scale bar = 100 μm.

On P10, we observed the lateral curvature of the medial pterygoid processes in both strains. However, these were more curved in the C57BL6J mice than in the BALB/cA mice (Fig 3B and 3E). The TVP angles between a line connecting the most inferior point with the superomedial point and a horizontal line were significantly different between the C57BL6J mice (74.3° ± 3.0°) and BALB/cA mice (105.2° ± 5.2°) (*P* < 0.01) (Fig 3G). The shapes of the SP still showed differences between the two strains (*P*< 0.05) (Fig 3A, 3D and 3H). Although the CSAs of the anterior TVPs showed no differences (*P* = 0.44) (Fig 3B, 3E and 3H), the CSAs of the posterior TVPs showed a considerable difference (*P* < 0.05) (Fig 3C, 3F and 3H). A muscle bundle was only identified inferior to the posterior TVP in the C57BL6J mice (Fig 3C).

### Comparable primordial muscles in the C57BL6J and BALB/cA mice

**Fig 4:**
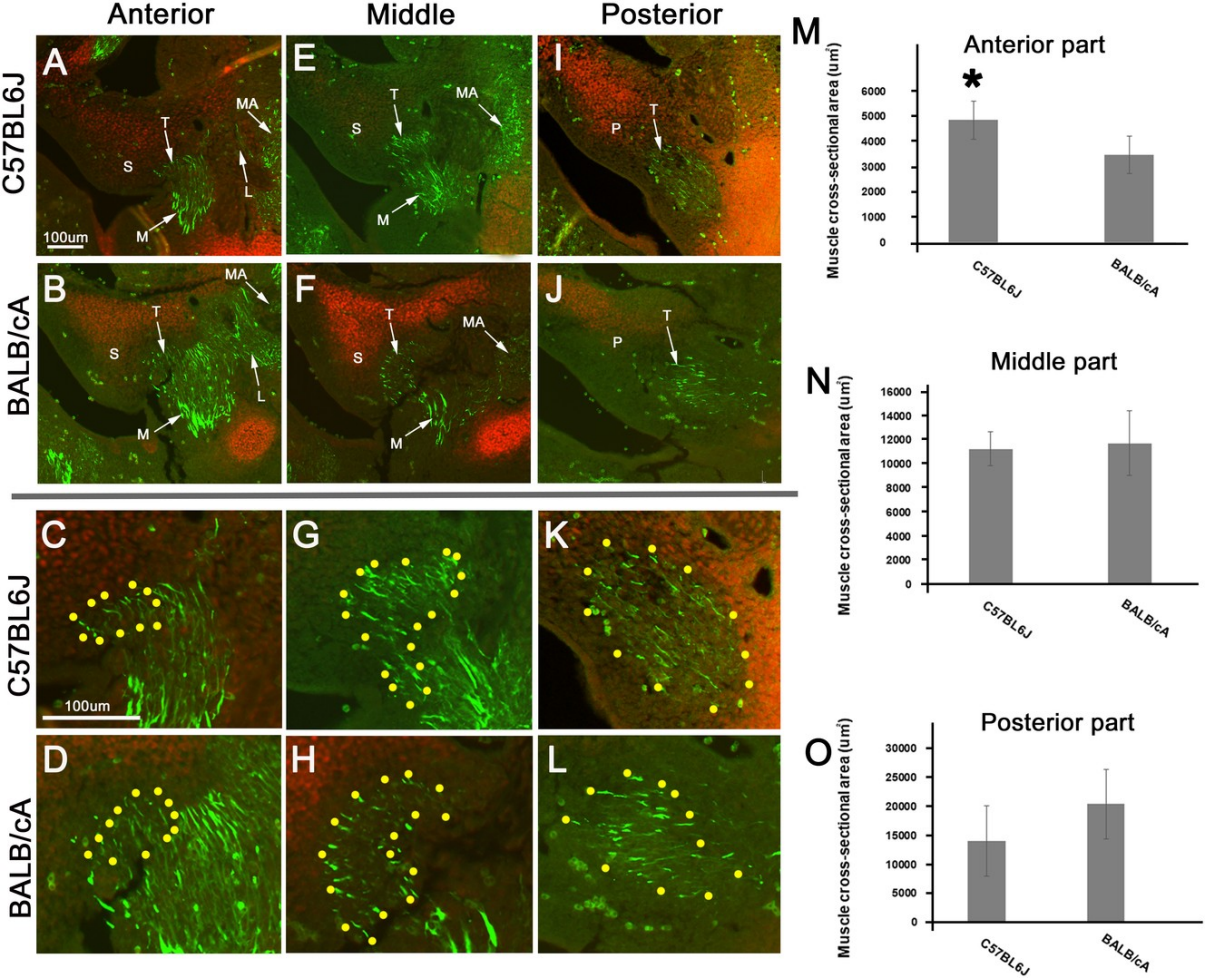
Comparable primordial muscles in C57BL6J and BALB/cA mice. Panels A, B, E, F, I, J show the frontal sections at E12.5. Panels C and D (G and H, K and L) show high magnification view of panels A and B (E and F, I and J). Yellow dots indicate anlage of the TVP (panels C, D, G, H, K, and L). Panels A, B, E, F, I, J (C, D, G, H, K, and L) show same magnification level. The TVP first appears medial to the medial pterygoid (M) at E12.5 (panels A, B, E, F, I, and J). The desmin-positive TVP (FITC) is located lateral to the Sox9-positive inferior spine (S) (Rhodamine) (panels A, B, E, F, I, J). The anterior TVP in the C57BL6J mice had a larger CSA than that in the BALB/cA mice (*P* < 0.05) (panel M). There were no differences in the primordial muscle size between the C57BL6J and BALB/cA mice in the middle (*P* = 0.79) or posterior (*P* = 0.24) parts of the TVP (panels N and O). L. lateral pterygoid; M. medial pterygoid; MA. masseter; S. inferior spine of the hypophyseal cartilage; T. tensor veli palatini; Scale bar = 100 μm.

The muscle-specific protein desmin was expressed in all muscle primordia, and Sox9 was expressed in most of the cartilage primordia (Fig 4). The desmin-positive TVP was located lateral to the Sox9-positive SP in this period. The anterior TVP in the C57BL6J mice had a larger CSA than that in the BALB/cA mice (*P* < 0.05) (Fig 4C, 4D and 4M). There were no differences in the primordial muscle size between the C57BL6J and BALB/cA mice in the middle (*P* = 0.79) or posterior (*P* = 0.24) parts of the TVP (Fig 4G, 4H, 4K, 4L, 4N and 4O).

### Development and growth of the TVP and age changes

**Fig 5:**
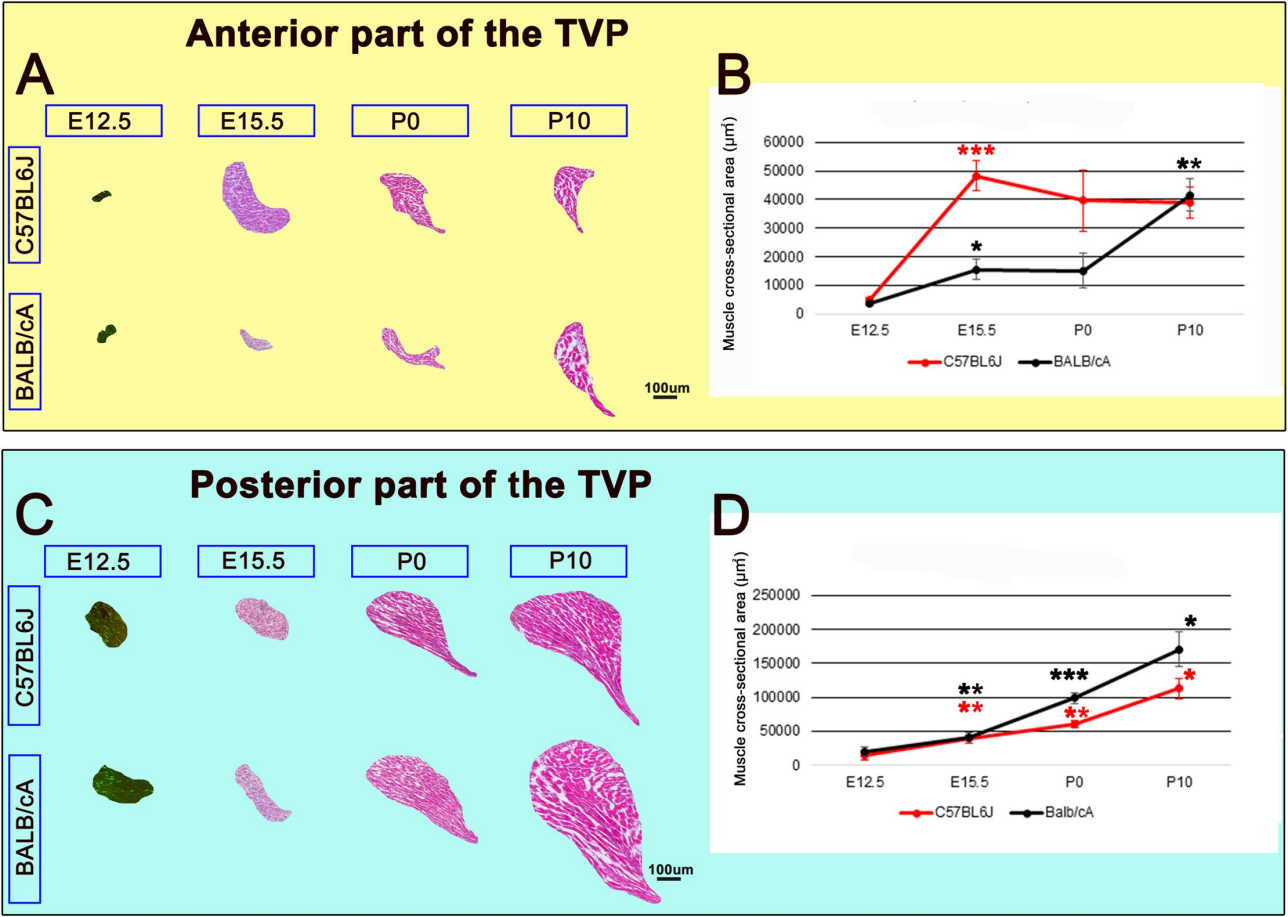
Development and growth of the TVP and age changes. Panel A (C) shows the cross sections of the anterior (posterior) TVPs form Figs 2–4. Panel B and D show the growth curve of the TVP. (Anterior part) The growth curve of the anterior TVP in C57BL6J mice is different from that in BALB/cA (panel B). (Posterior part) The posterior TVPs showed almost the same growth curve in the two strains between E12.5 and P10 (panel D). Scale bar = 100 μm.

In the C57BL6J mice, the anterior TVP significantly increased between E12.5 and E15.5 and subsequently showed a tendency to reduce between E15.5 and P10, whereas in the BALB/cA mice, it gradually increased between E12.5 and P10. Finally, both mice showed almost the same value at P10 (Fig 5A and 5B). Therefore, the anterior TVP in the C57BL6J mice exhibited a different growth curve than that in the BALB/cA mice (Fig 5B).

The growth curve of the posterior TVP was almost identical in the two strains, thereby showing a gradual increase between E12.5 and P0 (Fig 5C and 5D).

### Morphological association between the muscle and bone

**Fig 6:**
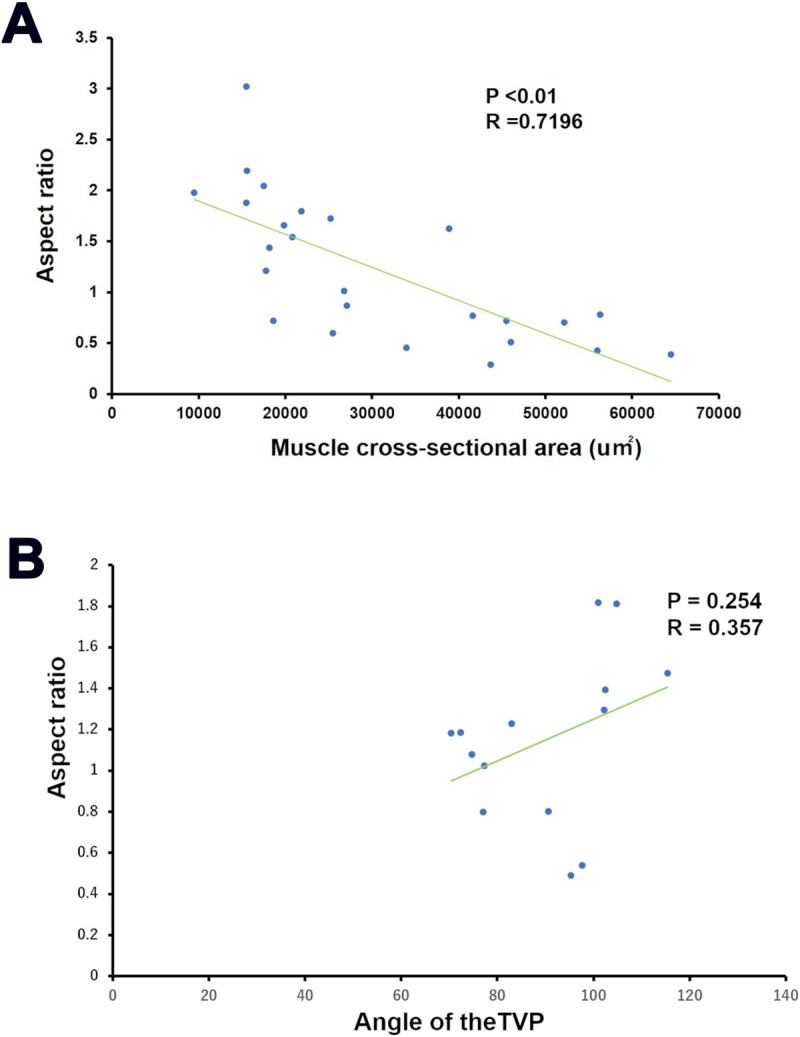
Morphological association between the muscle and bone in the C57BL6J, BALB/cA, and ICR mice. There are morphological correlations between the anterior TVP and the SP at E15 and P0 (R = 0.719, p = 0.008; panel A). However, there are not correlations between the TVP angle and the SP at P10 (R = 0.357, p = 0.254; panel B).

We examined a morphological correlation between the TVP and the SP in the C57BL6J, BALB/cA, and ICR mice. There were significant correlations between the CSA of the anterior TVP and the aspect ratio of the SP during E15 and P0 (R = 0.719, *P* = 0.008) (Fig 6A). However, there were not correlations between the TVP angle and the aspect ratio of the SP during P10 (R = 0.357, *P* = 0.254) (Fig 6B).

### Impact of bone deformation on muscle shape

**Fig 7:**
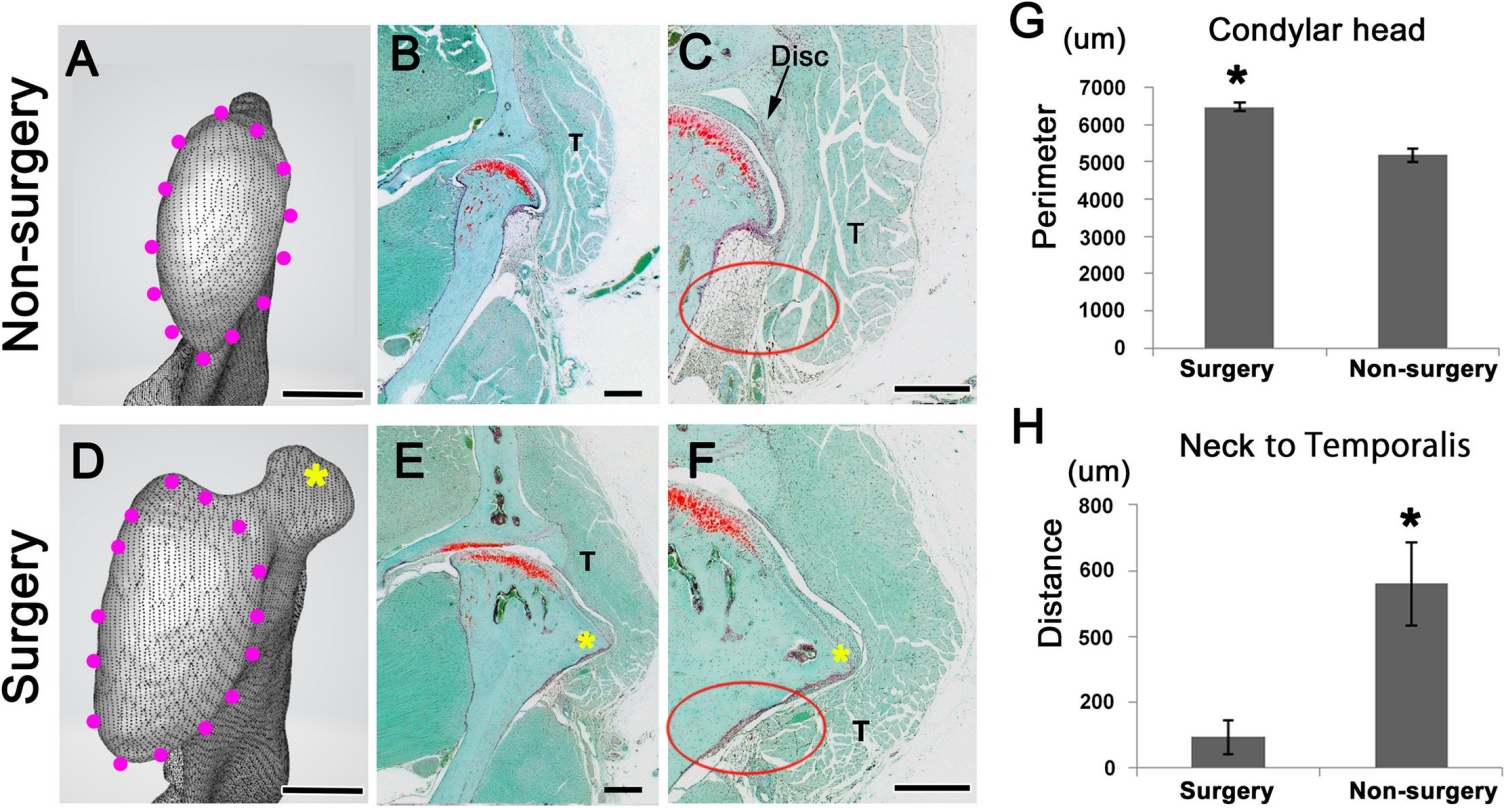
Impact of bone deformation on muscle shape. CT images show the top of the condylar head (panels A and D). Panels B and E show the frontal sections. Panels C and F show high magnification view of panels B and E. At 70 days after discectomy, deformation of the mandibular condyle is evident in the temporomandibular joints (panels A, D, and G). The shape of the temporalis muscle (T) has changed after discectomy (panels B, C, E, and F). The muscle on the discectomy side is not only in contact with the condylar head but is also in contact with the condylar neck (panels F and H; oval). The muscle on the non-surgery side is separated from the condylar neck (panels C and H; oval). Scale bar: 500 μm (panels A-F).

At 70 days after discectomy, the size of the mandibular condyle on the discectomy side was greater than that on the non-surgical side (Fig 7A and 7D). The perimeter of the top of the condyle on the discectomy side was larger than that on the non-surgical side (surgery side, 6479.21 μm; sham side, 5189.89 μm; *P* < 0.05) (Fig 7G). The posterior torus of the condyle was clearly observed on the sides subjected to discectomy (Fig 7D, 7E and 7F). The shape of the temporalis muscle had changed by discectomy (Fig 7B, 7C, 7E and 7F). The muscle on the discectomy side was not only in contact with the condylar head but also with the condylar neck (Fig 7F, oval). However, the muscle on the non-surgical side was separated from the condylar neck (Fig 7C, oval). The distance between the condylar neck and temporalis muscle on the non-surgical sides was larger than that on the discectomy sides (discectomy, 92.92 μm; sham surgery, 560.22 μm; *P* < 0.05) (Fig 7H).

## Discussion

Hallgrímsson et al. [3] reported differences between the skull shapes of A/WySnJ and aC57BL6J mice. A study by Kawakami and Yamamura [4] found that *M*. *spretus* mice have much thinner skulls than the C57BL6J, BALB/cA, C3H/HeJ, CBA/JNCr, ICR, or MSM/Ms mice. However, the underlying reasons for the differences in the skull shapes of different mice strains are not well characterized. Noden and Trainor [31] have described muscle–bone interactions during the embryonic period to cause different skull shapes. The neural crest cells, precursors of the skull, were shown to be located in the superficial layer of each pharyngeal arch and eventually wrap around the mesoderm-derived cells, which are the precursors of the head muscles. Although these cells are in close proximity to each other, few studies have investigated their intercellular interactions during the early developmental stages [32]. However, several studies have revealed the intercellular interactions between the skull and muscle anlagen after the fate of the cells is determined [33–35]. In this comparative anatomical study of C57BL6J with BALB/cA, we demonstrated the association between the shape of the muscles and bones in the craniofacial region. The effect of muscle development on the bone shape may represent an underlying mechanism of the evolution of skull phenotypic variations over several generations.

Several studies have investigated the mechanisms involved in the regulation of muscle mass. Denervation induces an immediate loss of skeletal muscle mass [36]. Although reinnervation after long-term denervation may result in the partial recovery of muscle mass, it is typically not restored to the original state [37]. A recent study showed that signaling by bone morphogenetic proteins (BMPs) strongly exacerbated the effects of denervation and fasting [38]. In addition, the authors concluded that BMP signaling, particularly Gdf5 (BMP14), was critical for the maintenance of muscle mass. Another study showed that BMP signaling pathway promoted skeletal muscle mass development and inhibited the wasting of denervated muscle [39]. In addition, the combined inactivation of Spry1 and Spry2 in the temporomandibular joint was shown to promote muscle mass growth and hinder bone formation, thereby suggesting that Spry1 and Spry2 may regulate the muscle size [40]. We showed that the anterior TVP in the C57BL6J mice was larger than that in the BALB/cA mice at E15.5 and P0. It is possible that BMP signaling and the activities of Spry1 and Spry2 may regulate muscle mass in these strains.

Epigenetics means modification in gene expression that does not involve alteration in the underlying DNA sequence. The control mechanisms have been grouped in three classes: (1) DNA methylation, (2) histone modification, and (3) ncRNA interaction [41]. DNA methylation is associated with the pluripotency and naive characteristics of stem cells [42], whereas DNA demethylation is the removal process of a methyl group from nucleotides, which is essential for cell fate decisions [43]. During the development of muscle stem cells, specific-myogenic factors are activated in a demethylation-dependent manner [44–46]. Histone acetylation, which is one of the histone modifications, is also a major change that affects gene transcription. Histone acetylation and deacetylation regulate many steps of myogenesis [47]. Histone deacetylases (HDAC) contribute to the molecular pathways and chromatin changes that regulate the tissue-specific gene expression during chondrocyte and osteoblast specification [48]. In this study, we demonstrated a morphological correlation between the muscles and bones during E15 and P0. If the bone volume reduces in size, then DNA demethylation seems to promote the expression of specific-myogenic factors. On the contrary, if muscle volume reduces in size, then HDACs might control the chondrocyte maturation by regulating the expression of matrix genes. Since epigenetic information can be inherited across multiple generations [41], there might be differences in the TVP shapes between two mice strains at E12.5. In addition, we demonstrated that there were not correlations between the TVP and the SP at P10. Therefore, epigenetic modification between the muscles and bones seem to rarely happen during postnatal period.

Unilateral partial discectomy induces dramatic changes in the condylar cartilage in the surgical TMJ [28]. A study by Xu et al. [28] showed chondrocyte clusters to be appearing at 8 weeks after discectomy. Afterwards, fibrillation was observed at 12 weeks. In contrast, unilateral partial discectomy was shown to trigger the degeneration of the articular cartilage in the contralateral sham-surgical TMJ of mice. Choen et al. [29] reported that contralateral sham-surgical TMJs remained the same at 4 weeks after surgery, whereas the increased proteoglycan straining was observed at 8 weeks after the surgery. An incision was made in the subcutaneous and muscle layers on the contralateral side (sham surgery) [28, 29]. However, we did not perform the sham-surgical operation in the contralateral TMJ. This study retarded the progression of articular cartilage degeneration in the non-surgical contralateral side. Therefore, we used the contralateral TMJ as the control.

After discectomy, the size of the mandibular condyle on the discectomy side was greater than that on the non-surgical side, and the shape of the temporalis muscle on discectomy side had changed. Therefore, inflammation seems to allow epigenetic modifications between the muscles and bones to occur. Recently, reduced skeletal muscle mass and function, namely sarcopenia, is related to low areal bone mineral density, osteoporosis [49]. A combined diagnosis of sarcopenia and osteoporosis has been defined, namely osteosarcopenia, that is associated with increased risks of gait and balance disturbances and of fracture in elderly men. However, it is not clear whether one condition has more of a causal influence than the other or if both conditions have an equal impact. Hence, there is growing interest in the interaction between muscle and bone.

The effect of muscle loading on the skull shape depends on the tendon–bone interactions [50]. To observe pure muscle–bone interactions without any tendon effects, we selected the area, wherein the muscle is in direct contact with the bone.
